# A microfluidic study of oil displacement in porous media at elevated temperature and pressure

**DOI:** 10.1038/s41598-021-99796-7

**Published:** 2021-10-13

**Authors:** Marzieh Saadat, Nora Birgitte Vikse, Gisle Øye, Marcin Dudek

**Affiliations:** Ugelstad Laboratory, Department of Chemical Engineering, Norwegian University of Science and Technology (NTNU), 7491 Trondheim, Norway

**Keywords:** Chemical engineering, Fossil fuels

## Abstract

Microfluidics methods offer possibilities for visual observations of oil recovery processes. Good control over test parameters also provides the opportunity to conduct tests that simulate representative reservoir conditions. This paper presents a setup and procedure development for microfluidic oil recovery tests at elevated temperature and pressure. Oil recovery factors and displacement patterns were determined in single- or two-step recovery tests using two crude oils, high salinity salt solutions and low salinity surfactant solutions. Neither the displacement pattern nor the recovery factor was significantly affected by the pressure range tested here. Increasing temperature affected the recovery factor significantly, but with opposite trends for the two tested crude oils. The difference was justified by changes in wettability alteration, due to variations in the amounts and structure of the acidic and basic oil fractions. Low salinity surfactant solutions enhanced the oil recovery for both oils.

## Introduction

Core flooding experiments are the classical way of performing oil recovery studies by displacing oil from saturated rock samples using various flooding approaches. An advantage of this method includes the possibility to perform measurements at elevated temperatures and pressures, i.e., similar to reservoir conditions. Even though most core floods are carried out at higher temperatures and pressures, there are limited investigations that have systematically varied these parameters. A few studies have shown that increasing temperature resulted in increased oil recovery by water flooding using pure water^1–3^. Low salinity water flooding is an enhanced oil recovery (EOR) method that has been employed to recover additional oil beyond the secondary recovery^4^. One study, where the pressure varied from 6 to 300 bar showed minor effects on the amount of oil recovered by low salinity water flooding^5^. Xie et al. observed that low salinity water flooding was more influenced by the water composition than temperature and pressure^6^. Combined low salinity and surfactant flooding has also shown positive effects on the oil recovery^7^. However, the method gave lower oil recovery when the temperature was increased from 23 to 90 °C, probably due to decomposition of the surfactant^8^.

Limitations of the core flooding method include long and not always repeatable measurements, insufficient number of core plugs from the reservoir and time consuming, specialized methods to visualize the processes inside the cores. The latter means that information about fluid displacement processes is normally based on indirect measurements. Immiscible fluid–fluid displacements can either be stable (i.e., high displacement efficiency) or unstable. The unstable displacement can further be categorized into viscous or capillary fingering, and are major reasons for inefficiency in subsurface two-phase flow^9^. To assess the displacement processes, it is useful to have thorough understanding of displacement stability and fluid flow pathways. Detailed mapping of fluid displacement in porous media can benefit from easy optical visualization. This, together with auxiliary measurements, can result in improved knowledge in the flow dynamics of fluids in pores and provide better input for numerical simulations, as shown recently by Yiotis et al.^10^.

Microfluidics is a method that has shown potential for visualization in oil recovery studies^11,12^, which can open a window into the black box of underground reservoirs. With the quicker performance, it can be considered a preliminary, alternative, or complementary method to core flooding studies. Examples of microfluidic EOR visualization studies include polymer^13,14^, surfactant^15^, alkaline surfactant polymer or foam^16,17^, nanocellulose^18^, low salinity surfactant^19,20^, and low salinity water flooding^21–23^. However, only a few studies have considered the effect of temperature on fluid displacement. One investigation used gas to displace heavy oil and followed the development of residual oil saturation on the walls of a square capillary at 55 and 85 °C^24^. The results showed good agreement with the computational fluid dynamics. Furthermore, they investigated the behavior of the residual oil at 200 °C, as the relevant temperature in steam injection applications, and showed that the remaining oil saturation decreased with increasing temperature. In another investigation, the steam assisted gravity drainage (SAGD) process was simulated using a micromodel as a reservoir with injecting steam^25^, and the oil recovery dynamics and the efficiency of an alkaline steam additive was studied. Finally, Wegner and Ganzer^15^ investigated the effect of salinity, surfactant concentration, injection rate and temperature (up to 50 °C) on oil displacement by surfactant solutions.

The objective of this study was to develop a microfluidic method to study oil displacement by low salinity surfactant solutions at higher temperatures and pressures. Two types of tests were used to follow the displacement and the amount of recovered oil: A) 1-step recovery tests, where microfluidic chips saturated with oil were flooded with high salinity brine at various temperatures and pressures. These tests were performed to determine the test conditions for the subsequent tests. B) 2-step recovery tests where the oil saturated chips were first flooded with high salinity brine followed by low salinity surfactant solutions (simulating EOR floods). The recovery factors were determined both at ambient conditions and at elevated temperatures and pressures.

## Results and discussion

### One-step recovery tests

#### Oil recovery at different pressures

**Figure 1:**
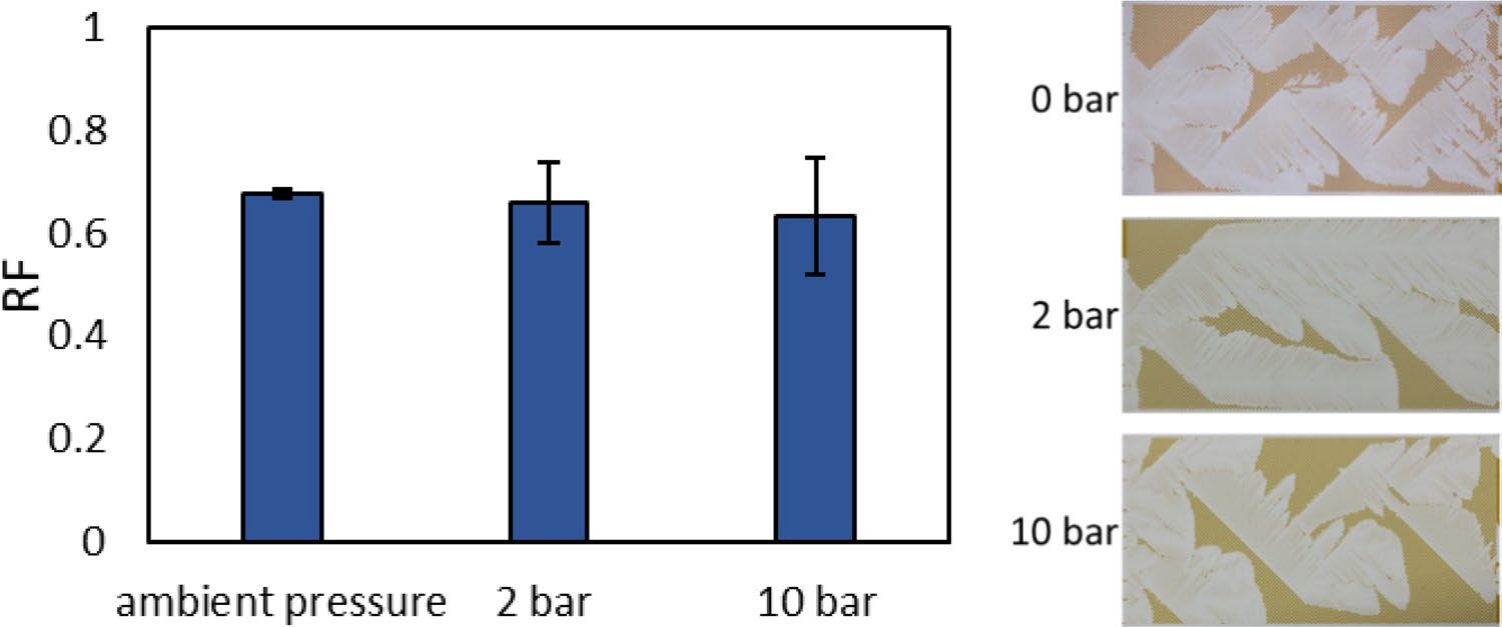
Recovery factors and patterns for crude oil A displaced by HS-Na at room temperature and different pressures (gauge). The change in color is due to different lighting.

To assess the effect of pressure on the recovery process, crude oil A was displaced by high salinity brine with sodium chloride (HS-Na) in different systems where the outlet is open to atmosphere or goes through pressure relief valves of 2 and 10 bar. Figure 1 shows the recovery factors (RF) and displacement patterns from the tests.

The oil saturation decreased between when the flood reached the network and breakthrough (i.e., when the flood reached the outlet). Having a lower viscosity fluid displacing a more viscous one in all three cases, viscous fingering occurred and the change in pressure did not affect the pattern. Unlike the measurements at ambient conditions, the pressure balance between the inlet and outlet was achieved with a noticeable delay after breakthrough at higher pressure measurements. This was most likely a consequence of higher number of connections and longer tubing (i.e., larger dead volume). The change in the oil saturation, however, was similar for all systems and occurred within the first couple of pore volumes of the flood. Although the standard deviation increased with pressure, the latter did not significantly affect the average recovery. This could be due to the importance of the pressure gradient in dictating the pattern and RF rather than the absolute pressure values on either side. Higher deviation in the results could have been caused by some differences in the experimental setup and procedure (e.g., type and number of connections). In another study^5^, core flooding tests conducted at 6 and 300 bar also showed the pressure did not affect the recovery significantly. Furthermore, the pressure did not affect the composition of the crude oil, as so-called “dead” (depressurized) crude oils were used in this work. Similar lack of the effect of pressure on coalescence between the different dead crude oils was observed in our previous work^26^. Based on these results, the pressurized tests were done at 2 bar for the rest of the study.

#### Oil recovery at different temperatures

**Figure 2:**
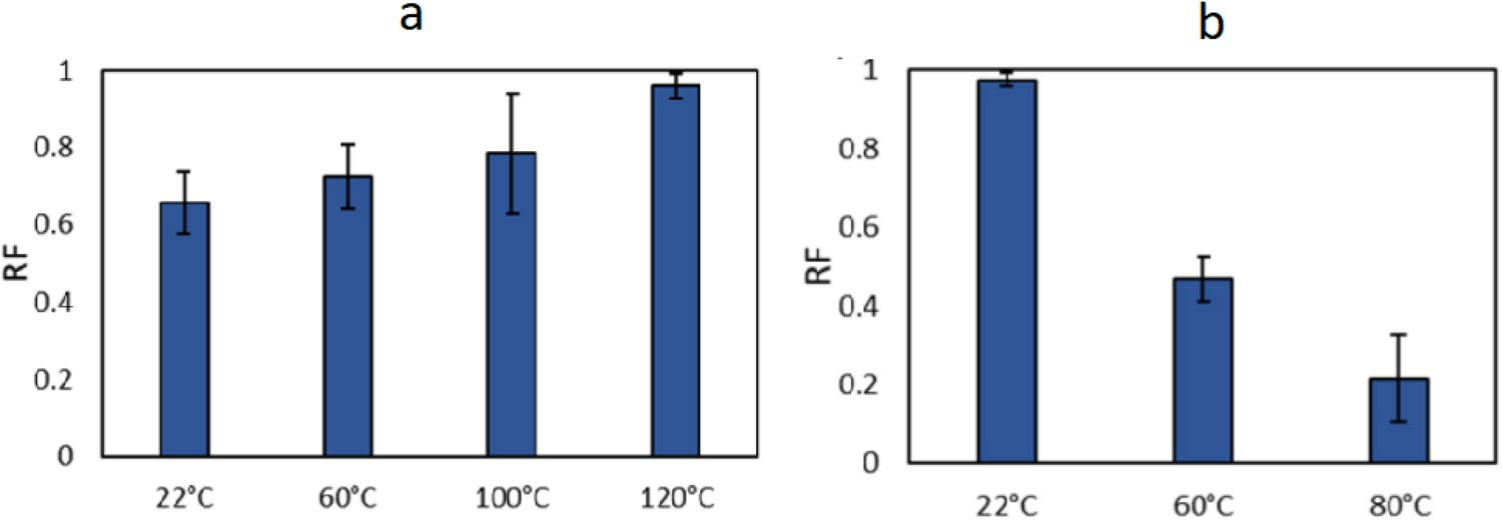
Crude oil A (**a**) and crude oil C (**b**) displaced by HS-Na at 2 bar and different temperatures.

To assess the effect of temperature on the oil displacement, experiments were conducted with the two crude oils at different temperatures at 2 bar. The recovery factors are presented in Fig. 2.

For crude oil A, the recovery went from partial recovery to almost complete recovery as the temperature increased from 22 to 120 °C, Fig. 2a. The corresponding displacement images are shown in supporting information (Figure S1). The standard deviation for the recovery tests at 100 °C were notably larger than the tests at both lower and higher temperatures. Repeated measurements, ten times in total to ensure that the deviation was not accidental or due to the operator error, showed displacement ranging from partial to almost complete recovery at this temperature (Figure S2). The large variation suggested that there was a transition from partial to almost complete recovery at this temperature. The recovery factor as a function of temperature for crude oil C is shown in Fig. 2b. In this case the recovery decreased from almost complete recovery at room temperature to low recovery at 80 °C. The corresponding displacement patterns are shown in supporting information (Figure S3).

The recovery trend for crude oil A was in agreement with core flood studies showing increased oil recovery by water flooding with increasing temperatures^1–3,6^. The decreasing recovery with temperature for crude oil C, however, was unexpected. The capillary number was considered in the further analysis:

$$Ca=\frac{{\mu }_{d}*v}{\gamma *cos\theta }$$

where µ_d_ and v are the viscosity and velocity of the displacing fluid, respectively, γ is the interfacial tension between the oil and water phases and θ is the contact angle with the surface. Both the viscosity and velocity of the aqueous (i.e. displacing) phase changed in the same way as a function of temperature in both cases. The interfacial tensions differed slightly between the two oils, but showed similar decrease when the temperature was increased, Table 1. This left differences in the wettability and wettability alterations as the plausible reason for the observed recovery trends.

**Table 1:** Interfacial tension values for crude oils A and C in HS-Na at different temperatures. *Measurements were done at two different temperatures to identify possible trends. 70 °C was the higher limit for the instrument, therefore 40 °C was chosen as an intermediate value.

Temperature	Interfacial tension [mN/m]	
	Crude oil A	Crude oil C
22 °C	20.4 ± 0.6	22.2 ± 0.6
40 °C*	15.8 ± 0.5	14.1 ± 0.4
70 °C*	17.3 ± 0.7	14.5 ± 0.1

**Figure 3:**
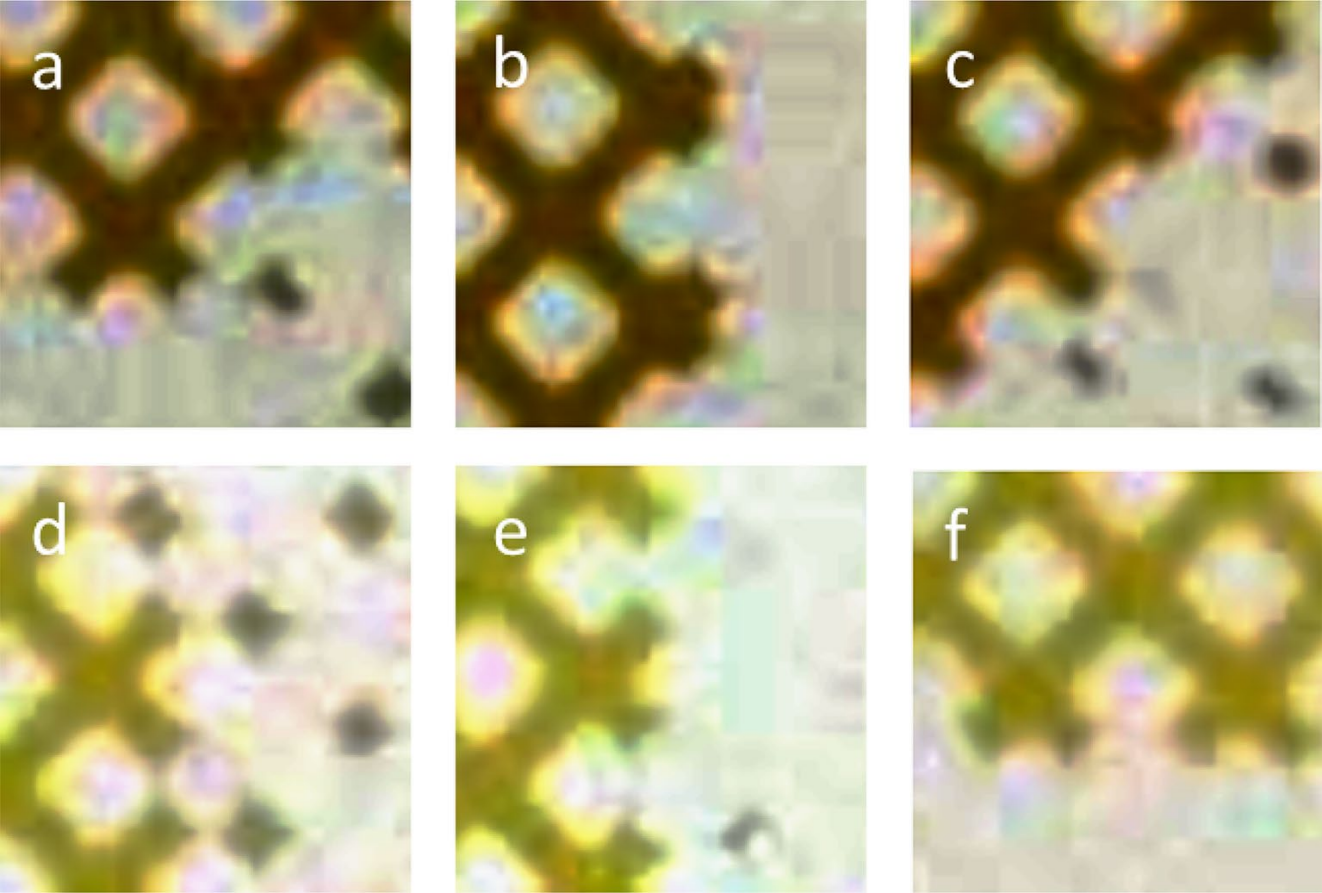
Close-up images with enhanced contrast of the border regions between the remaining oil and brine solution having displaced oil in the porous network for crude oil A at 22 °C (**a**), 60 °C (**b**) and 100 °C (**c**) and crude oil C at 22 °C (**d**), 60 °C (**e**) and 80 °C (**f**).

Figure 3 shows close-ups of the network at the oil–water border after being displaced by the aqueous solution (here with enhanced contrast, Figure S4 in SI shows the original snapshots). Considering the oil–water interface in the pore throats for crude oil A, the interface was curved towards the aqueous phase at 22 °C (Fig. 3a) and 60 °C (Fig. 3b), i.e. the pore throats were oil-wet in the vicinity of the interface. Due to the quality of the images, it was not possible to recognize changes in the extent of oil wetting when going from 22 to 60 °C. At 100 °C (Fig. 3c), however, the oil interface changed from concave to convex, meaning the pores appeared water-wet. This is also visible in the processed images with highlighted edges, where the oil interface at the highest temperature clearly becomes more rounded (Figure S5 in SI). The contrast between the phases was lower for crude oil C, which made observations more ambiguous, as seen in the original images in Figure S4. Nevertheless, based on the enhanced contrast images (Fig. 3d–f) and processed snapshots with highlighted edges (Figure S5d–f in SI), we suggest that the pore throats appeared slightly oil-wet and that the change in the curvature of the oil–water interface at increasing temperatures was less noticeable for crude oil C. This means that two factors could lead to the different recovery trends as a function of temperature: Different wettability after the bulk oil was displaced by aqueous solutions at room temperature for the two cases, and different extents of wettability alteration when the temperature increased. Crude oil A left the most oil-wet network after displacement at room temperature, but underwent a marked alteration towards water-wet network when the temperature increased. For crude oil C, the network was less oil-wet after displacement at room temperature and any change in wettability was less pronounced when the temperature increased.

The differences in wettability at room temperature and the response upon increasing the temperature must somehow be tracked back to differences in the chemical composition between the two oils, as the conditions otherwise were the same. Furthermore, the differences should be found in the interfacially active fractions of the oils, since these determine how the oils alter the wettability of the network. In a previous study involving crude oil C, it was demonstrated that both the basic and acidic fractions contributed to adsorption on a silica surface, but the basic fraction adsorbed in a higher amount^27^. It is possible that the higher amount of basic components in crude oil A and stronger interactions between basic components and the glass surface can justify more oil-wet conditions for crude oil A than for C at room temperature. This is supported by the thermodynamic modeling performed by Mansi et al., who showed that the adsorption of basic species at the oil/water interface is significantly reduced at higher temperatures, while it has less effect on acids^28^. Crude oil A, containing more basic components, could have been more susceptible to lower adsorption or desorption of basic species at the surface at higher temperatures, thereby explaining the larger wettability change towards water-wet networks. In contrast, crude oil C contained more acidic and less basic species, resulting in a less pronounced wettability change. Also other sources reported low effect of temperature on the adsorption of acids on surfaces^29^. Structural differences could affect the extent of desorption of various components from the glass surface (i.e. causing wettability alteration) and the partitioning of species into the water phase as a function of temperature. The latter was demonstrated by mixing the crude oils and HS NaCl water for 72 h at different temperatures. Table 2 shows that the aqueous phase had lower pH after being in contact with crude oil C than with crude oil A, due to more acidic components partitioning into the water phase. Furthermore, the pH decreased with increasing temperature, resulting in less dissociated silanol groups at the glass surface when exposed to the aqueous phase^30^. This could limit the desorption from the surface and thereby the wettability alteration as a function of temperature.

**Table 2:** HS-Na brine pH after mixing with crude oils A and C.

Temperature	Crude oil A	Crude oil C
22 °C	6.8	4.9
40 °C	6.9	4.6
80 °C	7.0	3.8

### Two-step recovery

**Figure 4:**
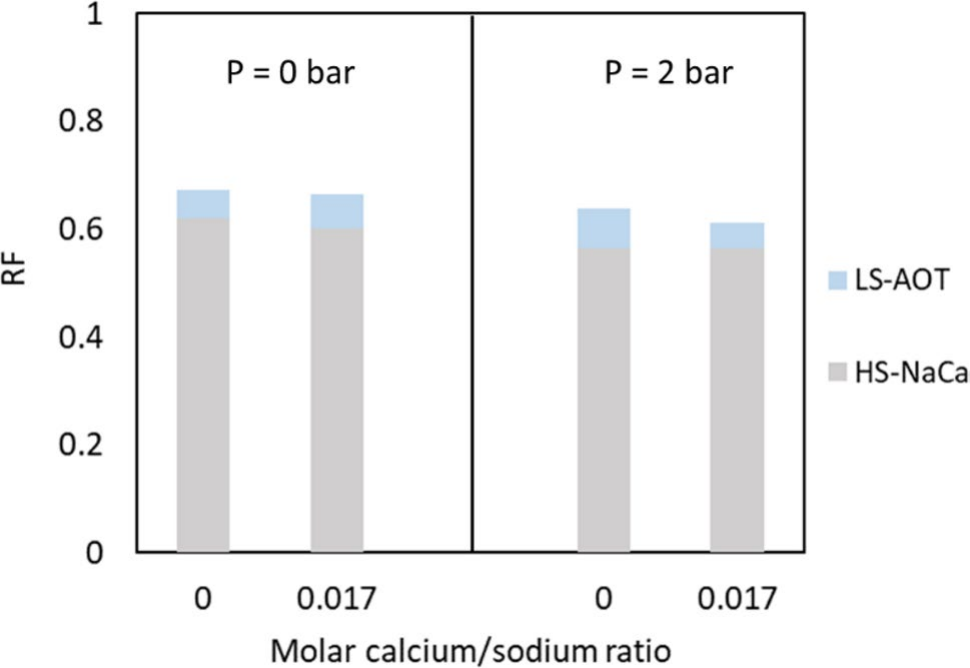
Average RF for crude oil A aged for 2 h and displaced first by HS-NaCa and then LS-AOT with and without calcium at 22 °C. Standard deviations for the measurements are within 10% error margin in all cases.

Two-step recovery tests involved aging of the oil saturated network and flooding, first with high salinity brine and then with low salinity brine containing AOT. In the high salinity flood with both sodium and calcium ions (HS-NaCa), the brine had both monovalent and divalent ions to better simulate secondary recovery. The subsequent low salinity surfactant flooding (LS-AOT) was done with and without calcium present. With calcium, the amount corresponded to optimal decrease in interfacial tension identified in a previous study^31^. Figure 4 presents the average RF for crude oil A at ambient pressure (left) and 2 bar (right) at 22 °C.

The oil recovery reached a plateau shortly after the breakthrough with the high salinity brine flooding, and the amounts were relatively independent of pressure. Furthermore, the low salinity surfactant flooding recovered comparable amounts of additional oil at both pressures. The flood recovered the remaining oil by releasing either the trapped oil droplets and ganglia in the flooded channels or off the boundary of the bulk of the oil like erosion. The oil recovery continued as long as the flood was injected. The presence of calcium did not have any consistent effects on the recovery. At atmospheric pressure, the recovery was 1% higher with calcium in the low salinity brine. At 2 bar, however, the additional recovery was 7.4% without and 4.8% with calcium in the low salinity surfactant solutions. Capillary desaturation curves have shown that increasing the capillary number results in lower residual oil saturation^32^. One way of increasing the capillary number is to reduce the interfacial tension between the oil and aqueous phase. For crude oil A the interfacial tension was reduced from 13.3 mN/m against the high salinity solution (HS-NaCa) to 0.4 mN/m against the low salinity surfactant solution (without calcium), and it was clear that this resulted in increased oil recovery.

**Figure 5:**
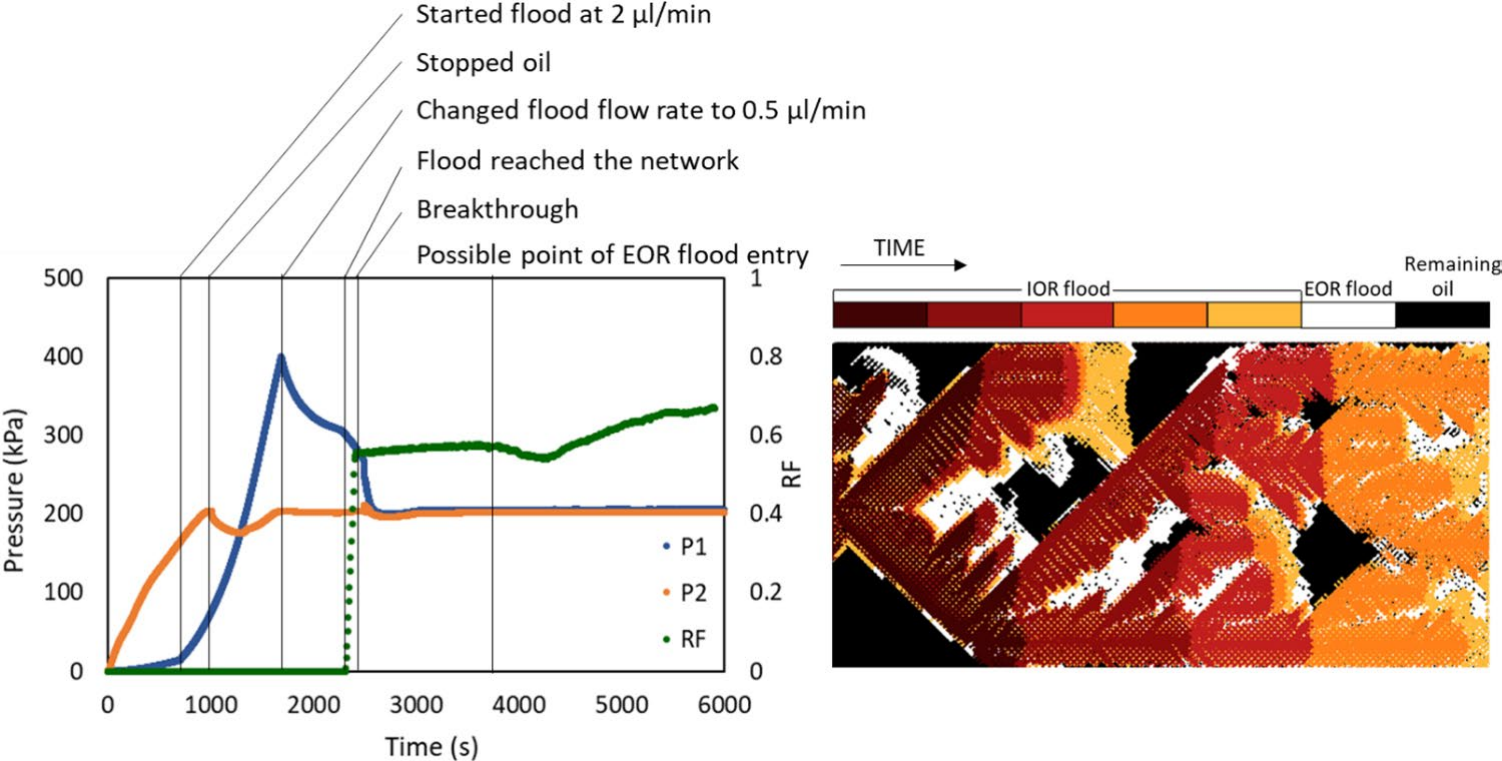
Dynamic pressure and recovery factor (left) and a time-lapse of the displacement pattern (right) for crude oil A aged for 2 h and displaced by HS-NaCa followed by low salinity surfactant at room temperature and 2 bar.

Figure 5 shows the dynamic recovery of crude oil A in terms of pressure, recovery factor and displacement pattern. The pressure plot shows how the pressure on the inlet side (P1) and the outlet side (P2) changed from the moment the oil pump was started at 2 µl/min. P2 increased, but P1 lagged due to the high viscosity of the oil at room temperature. The water pump was started at 2 µl/min after oil entered the inlet tube. As a result, P1 increased with a higher and steady pace. The pressure in the outlet showed a dip when the oil pump was stopped, but recovered due to the balance from the water pump. The oil kept progressing in the inlet tube until P1 and P2 were equal, but the flow of oil was reversed towards the outlet once P1 surpassed P2. The flow rate was changed to 0.5 µl/min when the oil in the inlet tube approached the inlet and the pressure started to decline. The rate of decline slowed down before the flood reached the network, due to the capillary pressure. At this point oil started to be recovered and the RF (green points) quickly increased. Most of the change happened within the first pore volume of the flood after its introduction to the network and the oil recovery slowed down shortly after breakthrough. The pressure also declined rapidly towards P1 upon breakthrough and both P1 and P2 remained at 2 bar for the rest of the experiment. The low salinity surfactant solution was added after about 4000 s. A small (artificial) decrease in the oil recovery was seen at the same time and was due to remaining oil in the inlet channels (not in the field of view and therefore not covered by image analysis) that was released due to the presence of surfactant. Since the extraction of oil occurred at a slower rate, the oil saturation appeared to increase for some of the analyzed images, resulting in lower RF. The recovery increased again shortly after. This artefact did, however, not happen frequently. The time-lapse image demonstrates the progress of the water phase displacing the oil phase. Starting from dark red to orange shows the flood towards breakthrough, yellow represents the state of the chip at the end of high salinity flooding and white corresponds to the recovery by the low salinity surfactant flood.

**Figure 6:**
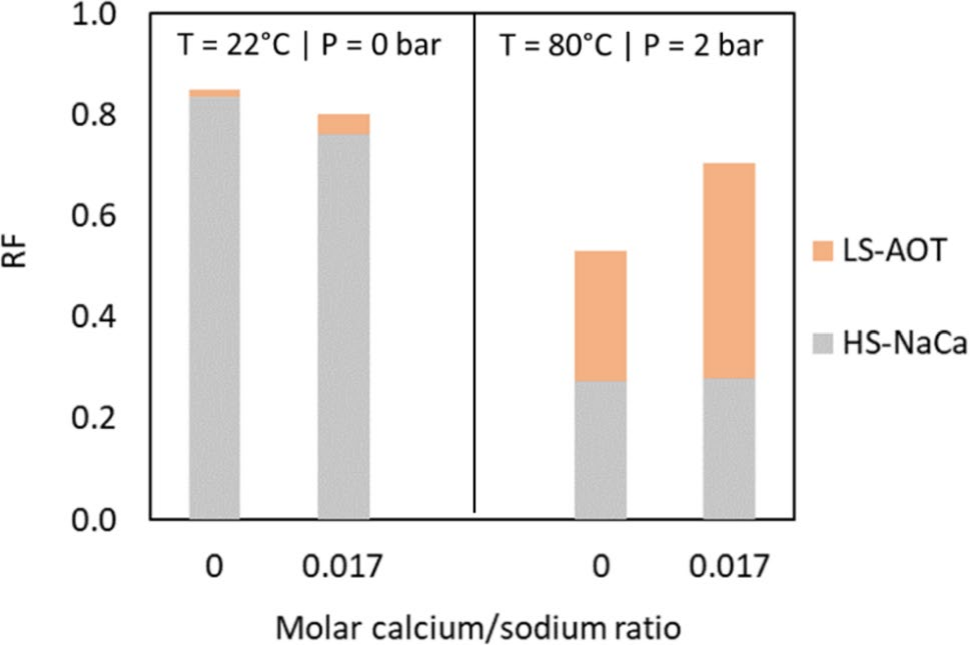
Average RF for crude oil C aged for 2 h and displaced first by HS-NaCa and then the different EOR fluids. Standard deviations for the measurements are within 10% error margin in all cases.

Figure 6 shows the RF for crude oil C at ambient conditions and at elevated temperature and pressure after displacement with high salinity brine followed by low salinity AOT. As in Fig. 2, the RFs after high salinity flooding were significantly reduced with increasing temperature from 22 to 80 °C. However, the RF increased significantly after low salinity surfactant flooding at the higher temperature. The interfacial tension between crude oil C and HS-NaCa and LS-AOT was 15.10 and 0.21 mN/m, respectively, and the capillary number was 2 orders of magnitude larger for the low salinity surfactant solution than for the high salinity solution, which can account for higher recovery. Furthermore, the presence of calcium in the low salinity solutions enhanced the oil recovery at both ambient and elevated test conditions. At ambient conditions, the low salinity surfactant solution with calcium recovered 4.1% additional oil (compared to 1.4% additional oil without calcium). At elevated temperature and pressure, the presence of calcium improved the low salinity surfactant recovery by 14%. Previously, it has been shown that calcium can increase the interfacial activity of AOT^33^, which can explain this effect. At similar ionic strength, the presence of calcium can modify the orientation of AOT at the oil–water interface or even improve its packing parameter, which would lead to more surfactant molecules at the interface, hence lower interfacial tension and possibly higher oil recovery.

**Figure 7:**
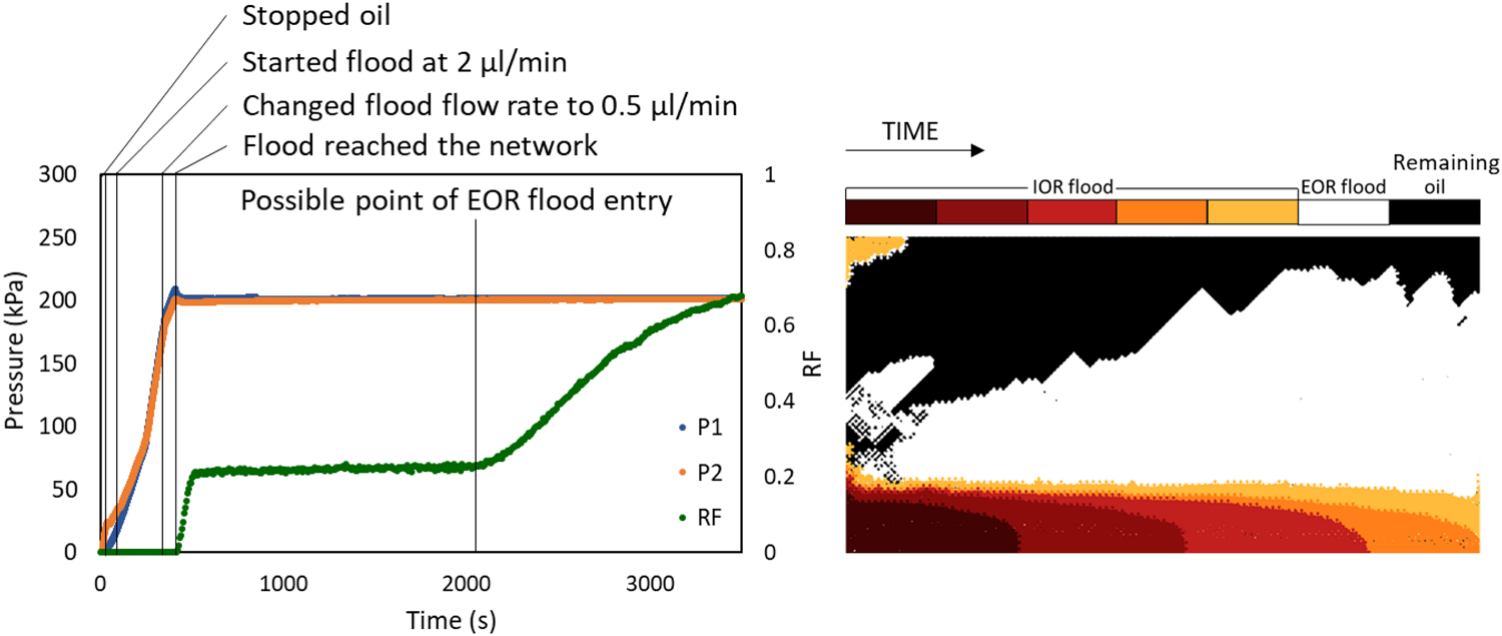
Dynamic pressure and RF (left) and a time-lapse of the displacement pattern (right) for crude oil C aged for 2 h and displaced first by HS-NaCa and then LS-NaCa AOT at 80 °C and 2 bar.

The dynamic recovery and pressure as well as the evolution of the displacement pattern for crude oil C displaced by high salinity brine followed by low salinity AOT solution at 80 °C and 2 bar is presented in Fig. 7. In contrast to what was seen for crude oil A, the pressure equilibrated quickly between the inlet and outlet due to the lower viscosity of the oil. In other words, the required pressure needed to push the oil in the tubing and microfluidic chip was much lower for oil C. For the same reason, the inlet pressure did not increase much above 200 kPa and the displacement occurred at a few kPa pressure gradient. As expected, the major change in oil saturation happened within the first injected pore volume of the flood, between reaching the network and breakthrough. Afterwards, most of the tests reach a plateau, while some showed a slow decrease in the oil saturation as explained above. Once the surfactant solution reached the network, however, the recovery accelerated (around 2000s in Fig. 7). Unlike the lower temperature tests, the surfactant solution did not release the oil droplets trapped inside the pores, but only pushed the bulk of the oil towards the outlet. When > 60% of the oil was recovered, the remaining oil consisted only of disconnected patches of oil ganglia and droplets and a recovery plateau was reached again. The time-lapse image for the same test demonstrates the progress of the water phase displacing the oil phase. Starting from dark red to orange shows the breakthrough, yellow shows the state of the chip at the end of high salinity flooding, and white presents the significant change in saturation by the low salinity surfactant flood. In agreement, Kenzhekhanov also showed the continued recovery of oil by the surfactant flood beyond breakthrough at both 20 °C and 80 °C, while the brine flood almost did not recover additional oil after breakthrough in either temperature^34^.

## Conclusion

The development of a microfluidic setup and procedures for visualization of oil displacement in porous media at elevated temperature and pressure was presented. The versatility of the method was demonstrated in single- or two-step recovery tests where crude oils were displaced by high salinity brines and low salinity surfactant solutions, and recovery factors and displacement patterns were determined. It was shown that pressure up to 10 bar did not significantly affect the recovery or displacement patterns. The temperature, on the other hand, clearly affected the recovery. The trend in recovery with increasing temperature was opposite for the two investigated crude oils. This was attributed to differences in the amounts and structure of the acidic and basic components in the oils, resulting in different wettability conditions as a function of temperature. In the two-step recovery tests, the second flood comprising low salinity surfactant solutions increased the oil recovery for both oils. Overall, the developed method has a potential to be used as a screening method and could greatly decrease the time and effort spent on performing multiple core flooding experiments.

## Methods

### Microfluidic setups and procedures

**Figure 8:**
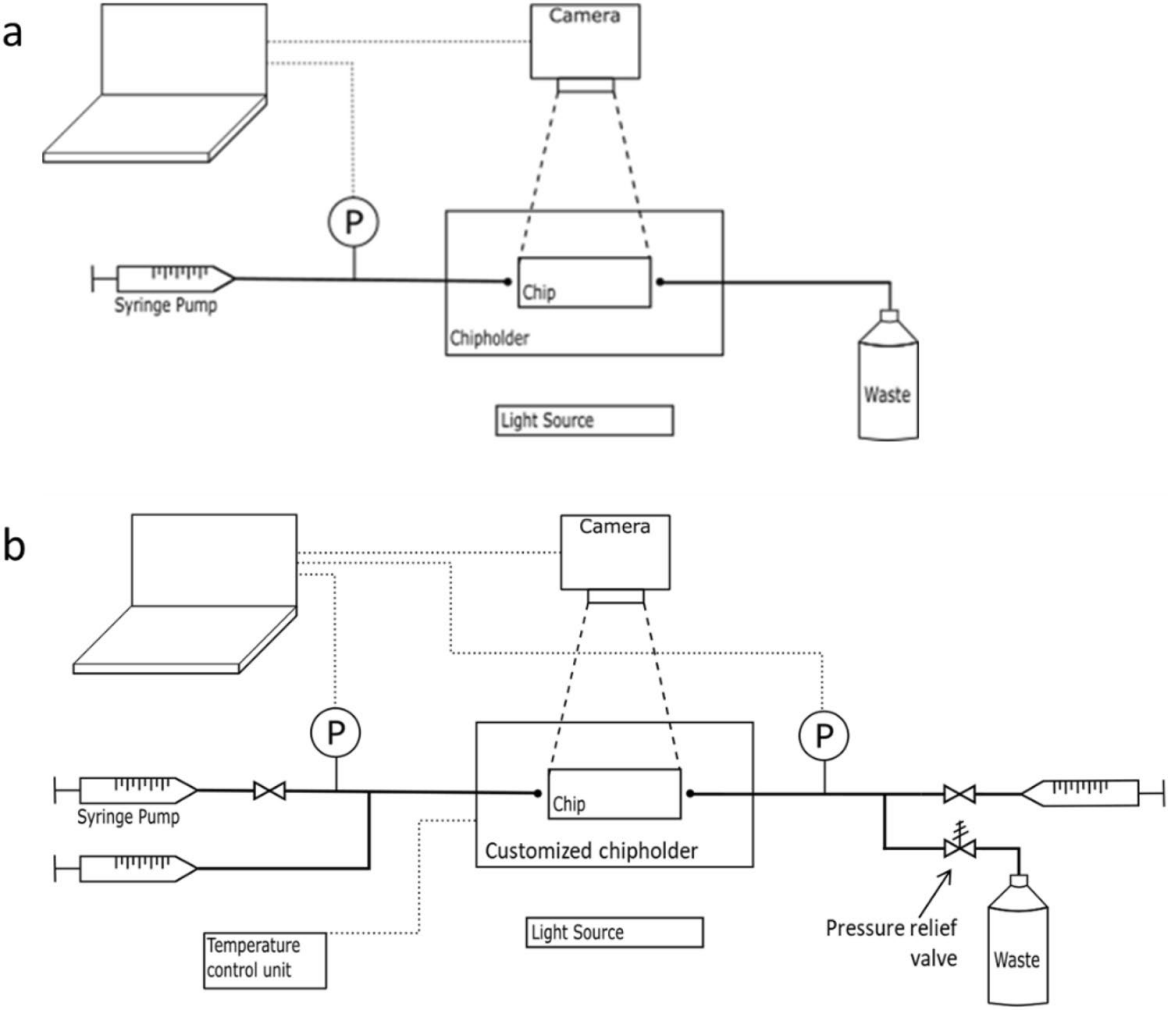
Schematic illustration of the microfluidic setup for tests at ambient conditions (**a**) and at elevated temperatures and pressures (**b**). The dimensions shown are not to scale^21^.

1-step and 2-step recovery tests at ambient conditions were conducted utilizing the same setup developed in a previous study^21^. The setup consists of a flow unit, imaging equipment, pressure sensor, chip holder, and micromodel (Fig. 8a). Uniform network micromodels (Micronit Microtechnologies) were used as models for the porous rock. The channels were 50 µm wide and 20 µm deep and were interconnected in a network area of 1 cm × 2 cm. The chips were made of borosilicate glass and were hydrophilic. The internal volume of the network area was 2.1 µl, and the porosity was 0.52. For 1-step recovery at ambient conditions, the chip was first saturated with a crude oil. Subsequently, the inlet tube, pre-filled with the flood solution, and the outlet tube were connected to the chip through the chip holder. The fluids were pumped at 0.5 µl/min and the total volume of a single flood was 10 µl (4.8 PV). For the 2-step recovery tests, the oil-saturated chip was aged in a custom-made aging holder at room temperature for 2 h (see Sect. "Fluids"). The chip was first flooded with high salinity brine followed by low salinity surfactant solution at the same flow rate. The inlet tube was detached after the high salinity flood and the line was filled with the secondary flood before it was reconnected and pumped. Each test was repeated at least three times to verify the repeatability.

**Figure 9:**
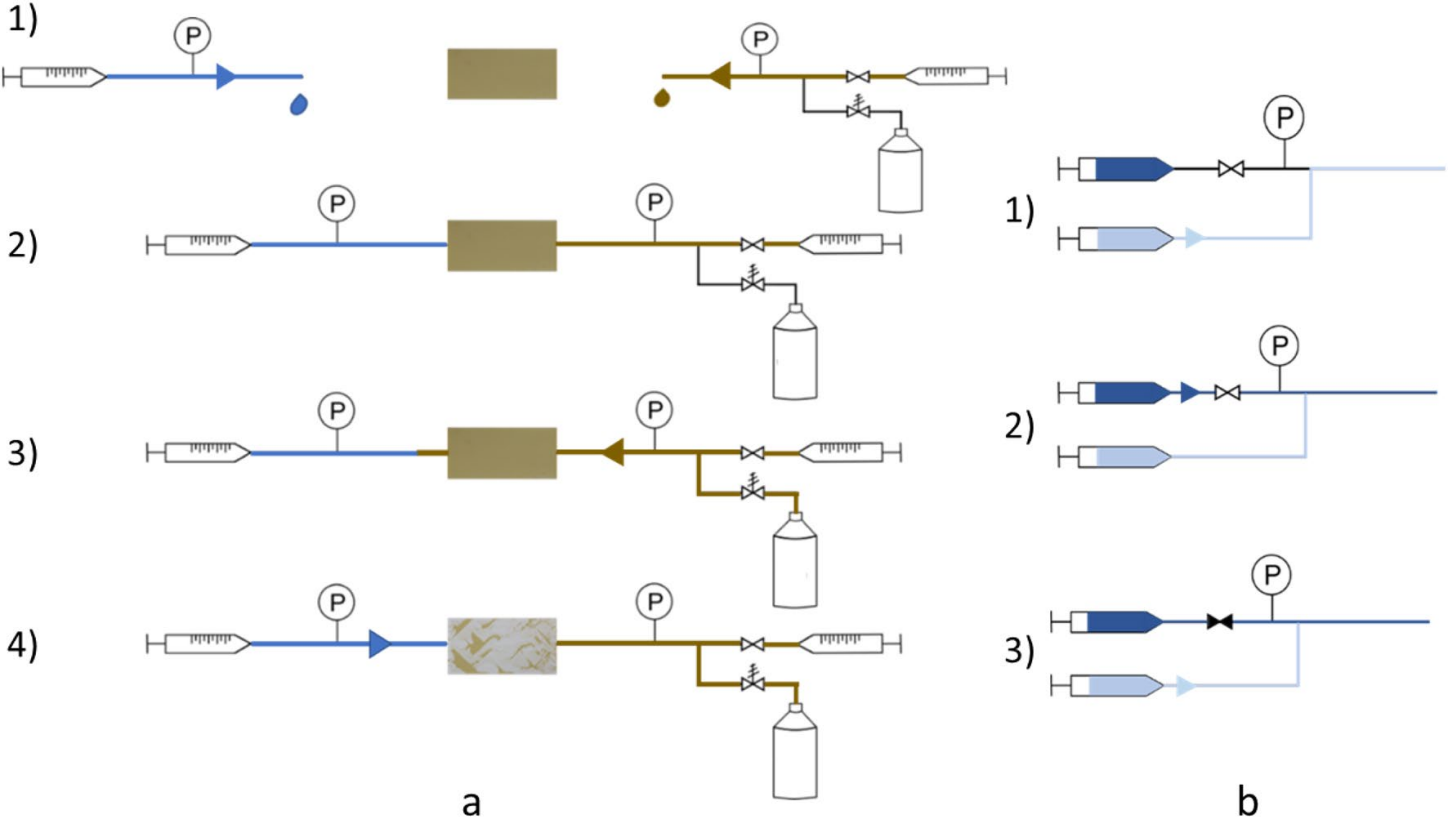
(**a**) 1-step recovery test procedure: 1) Filling the inlet and outlet lines with HS-Na and crude oil, respectively; 2) connecting the tubes and the oil-saturated chip; 3) pumping oil at 2 µl/min; 4) pumping water initially at 2 µl/min and later at 0.5 µl/min. (**b**) Procedure for filling the water line in 2-step recovery test: 1) flow the second flood; 2) flow the high salinity brine; 3) shut the valve on the high salinity brine line and use the second flood syringe for injection after connecting to the setup.

Several modifications were made to the setup to enable displacement studies at elevated temperature and pressure. A customized chip holder (see details below) was attached to a temperature control unit, while a pressure relief valve was placed on the outlet line and set to the desired pressure, Fig. 8b. The principle of this configuration was to pump the oil from the outlet tube towards the inlet side to increase the pressure before the flooding started. Due to the pressure relief valve, the pressure in the outlet line did not increase passed the set pressure (2 or 10 bar in our experiments) and was kept constant throughout the experiments. Figure 9a illustrates the procedure for preparing the 1-step recovery tests. For a two-phase displacement, it is crucial to avoid air in the system before the parts are connected. Therefore, the chip and outlet tube were filled with oil, while the inlet tube was filled with the aqueous flood (Fig. 9a, step 1) prior to connecting the tubes via the holder (Fig. 9a, step 2). Initially, oil was pumped at 2 µl/min and, as the pressure in the outlet increased, oil moved into the inlet tube to equilibrate the pressure between the two sides (Fig. 9a, step 3). After the inlet pressure surpassed half of the target pressure, the flood pump was started at 2 µl/min (Fig. 9a, step 4). Shortly after, the oil pump was stopped and the valve was shut. The rate of the flood pump was changed to 0.5 µl/min when the pressure approached the set pressure or before the flood reached the end of the inlet tube, whichever came first.

Filling the flood line was more complex for the 2-step recovery tests. Due to pressure fluctuations, two pumps could not be used during the tests because stopping one would cause a plunge and starting the other would increase the pressure suddenly. Therefore, an arrangement was made to pump both floods using one pump. Two syringes with flooding fluids were connected to the inlet by a tee connection, Fig. 9b. To avoid trapping air in the line, the tertiary flood was pumped until the line was filled (Fig. 9b, step 1). Then, the high salinity flood was pumped so that the inlet tube up to the tee was filled with the high salinity brine (Fig. 9b, step 2). The valve on the high salinity syringe line was then shut. This way, with only pumping the second flood syringe, the chip was flooded with both fluids (Fig. 9b, step 3). The length of the tube between the tee and the sample determined the volume of the high salinity flood. For a tube length of 5 cm the flood was calculated to be 10 µl. Even though some mixing (driven by diffusion) could occur at the contact points between the two floods, as later seen during the experiments, a steady-state recovery was quickly reached within the first PV of the high salinity flood. Consequently, any mixing between the floods would not have a large impact on the outcome of the 2-step recovery measurements. Then, the flood line, outlet tubing and the aged (2 h at room temperature) oil-saturated chip was connected through the customized chip holder. The rest of the test procedure was similar as described for the 1-step recovery tests.

After each experiment, the chip underwent a cleaning procedure to remove any residues from the network. The solvents used to flood the micromodel were xylene, isopropanol, and de-ionized water. The steps dissolved and removed any remaining oil and salt in the network. Finally, the chip was heated in a programable ashing furnace at 475 °C to dry and ensure disintegration of any organic remains.

### Imaging

Imaging started just before the flood reached the network. Images were automatically taken every 6 s by a high-resolution camera (Canon EOS 90D), fitted with a macro lense. They were processed using ImageJ by color thresholding as well as adjusting the saturation and brightness. The process converted the color images to 8-bit black and white ones where the oil was represented by black and the rest of the image was in white (glass network and water phase). Using the region of interest (ROI) manager tool, the white or black surface area can be extracted in pixels or as a percentage of the whole area. The oil recovery was then calculated based on the difference in oil saturation between the initially oil-filled chip and a flooded one. The recovery factor (RF) was defined as the ratio of extracted oil to original oil in place. Dynamic change in RF was also calculated using the same procedure on the whole image sequence taken during the test. The number of pore volumes injected for each image was calculated using the injection flow rate, capture time of the image and the volume of the pore network (2.1 µl). To create the time-lapse pictures, a group of selected images were processed as described above and then compiled using Gimp.

### Aging holder

The aging holder was used to avoid getting air in the chips, and therefore maintain two-phase displacement, and also prevent oil evaporation during aging. It was designed to block the inlet and outlet ports of the chips using plugs and was made of stainless steel. The chip slots were covered with a silicone mat to avoid breaking the micromodels. The aging time was optimized to 2 h in a previous study^21^, while the effect of temperature was considered here. 1-step displacement tests showed that aging decreased recovery by 7–14% in a range from room temperature to 100 °C, Figure S6. However, the differences between temperatures were not significant and 2 h at room temperature were used as the standard aging conditions.

### Design of chip holder for elevated temperature and pressure

**Figure 10:**
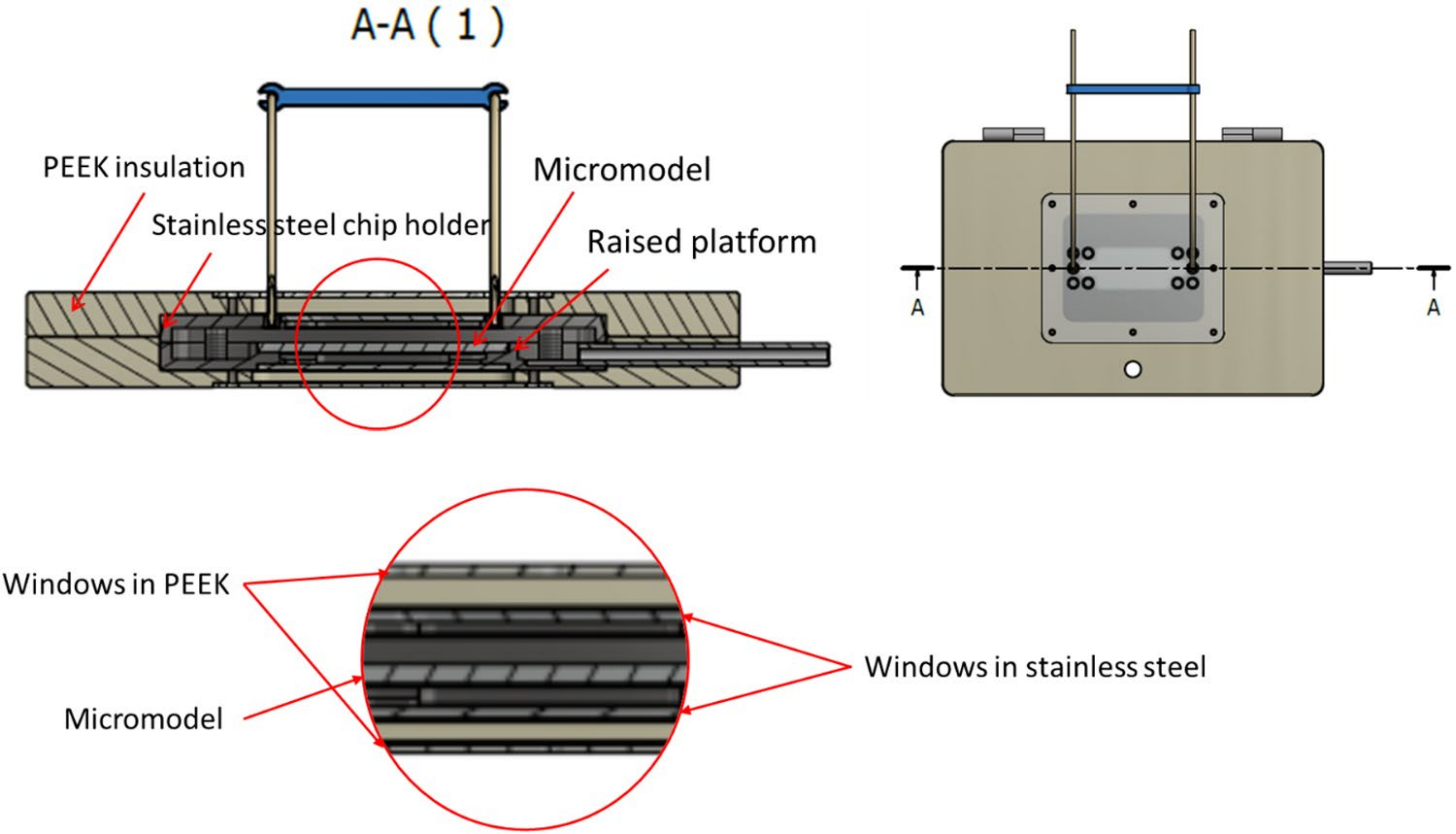
The high temperature—high pressure (HTHP) chip holder design. The hatched area of the cross section shows where there is material, and the rest showing the open space for air circulation. Technical drawing courtesy of Robert Karlsen, Engineer at the Mechanical Engineering Workshop at NV Faculty, NTNU.

Experiments at elevated temperature and pressure required a chip holder that could withstand more extreme conditions (Fig. 10), which is an improved design of a holder used in a previous study^35^. Threaded screw-in attachment fittings (10–32 UNF with PEEK/steel ferrule assembly) were used to connect the tubing (PEEK, 1/16’’) to the chip and withstand the pressure in the system. The inner core of the holder was made of stainless steel with two glass windows on top and bottom to allow light passage and quality images. The stainless-steel core also housed the heating elements and the temperature sensor that were connected to and controlled by the temperature control unit. It also contained a raised platform to keep the micromodel vertically lifted with enough space above and beneath the glass surface for hot air circulation and uniform heating of the sample. During initial tests, the temperature shown by the control unit was validated with an external temperature probe. The perimeter of the raised platform was covered with a rubber cushion to avoid direct contact of the sample with steel, to avoid temperature gradients and to avoid breaking the chip in case of excess pressure from screwing in the fittings. The gap between the chip and the metal top cover was made so that the holder also could be used with ferrules for attaching the inlet and outlet tubing to the chip for lower pressure tests. Lastly, the metal holder was placed in a PEEK box covering the exposed metal parts, both for safety and avoiding burns, and as an insulation to lower the heat loss. The PEEK cover also had two glass windows on the top and bottom that aligned with the metal holder windows to allow light to travel through for proper imaging.

### Fluids

Two crude oils from the Norwegian Continental Shelf were used for the measurements. Their properties are presented in Table 3. Different low salinity surfactant solutions were prepared as the displacing fluids, containing either sodium chloride or both sodium chloride and calcium chloride. Sodium dioctyl sulfosuccinate (AOT) was used as the surfactant. The displacing fluids are listed in Table 4.

**Table 3:** Crude oils properties. Viscosity data for 20 °C. Both oils were used in previous reports of our group and the naming convention is kept for consistency^36^. TAN and TBN are the total acid and base numbers, respectively.

	API [°]	Viscosity [mPa*s] @20 °C	TAN [mg KOH/g oil]	TBN [mg KOH/g oil]	SARA [% wt.]			
					Saturates	Aromatics	Resins	Asphaltenes
Crude oil A	19.2	354.4	2.2	2.8	50.6	31.2	15.7	2.5
Crude oil C	23	74.4	2.7	1.1	64.9	26.3	8.4	0.4

**Table 4:** Displacing fluid properties.

Flood name	Abbreviation	Brine concentration (M)	Ca/Na (mole/mole)	Viscosity (mPa*s)
High salinity brine	HS-Na	0.6	0	1.14
High salinity brine	HS-NaCa	0.6	1/50	1.09
Low salinity brine + surfactant	LS-Na AOT	0.02	0	1.06
Low salinity brine + surfactant	LS-NaCa AOT	0.02	1/50	1.03

### Characterization

The interfacial tension between oil and water phases were measured with a spinning drop tensiometer (SVT 20 N, DataPhysics Instruments).

The viscosity of the two crude oils were measured at different temperatures. Rheometer Physica MCR 301 (Anton Paar) was utilized to conduct the measurements. The results are shown in Table S1 (Supporting Information).

## Supplementary Information

Supplementary Information.
