# Supplementary material for: A microfluidic study of oil displacement in porous media at elevated temperature and pressure
Source: Sci Rep. 2021 Oct 13;11:20349. doi: 10.1038/s41598-021-99796-7 (PMC8514519; doi:10.1038/s41598-021-99796-7)
Supplement: Supplementary file 1 — Supplementary Information. [file 41598_2021_99796_MOESM1_ESM.docx]

**Supporting information**

Table S1. Viscosity measurements for crude oils A and C at different temperatures.

| Temperature (°C) | Crude oil A Viscosity (mPa*s) | Crude oil C Viscosity (mPa*s) |
| --- | --- | --- |
| 80 | 16 | 6 |
| 70 | 23 | 9 |
| 60 | 35 | 12 |
| 50 | 56 | 17 |
| 40 | 95 | 26 |
| 30 | 173 | 41 |
| 20 | 354 | 74 |
| 10 | 864 | 135 |


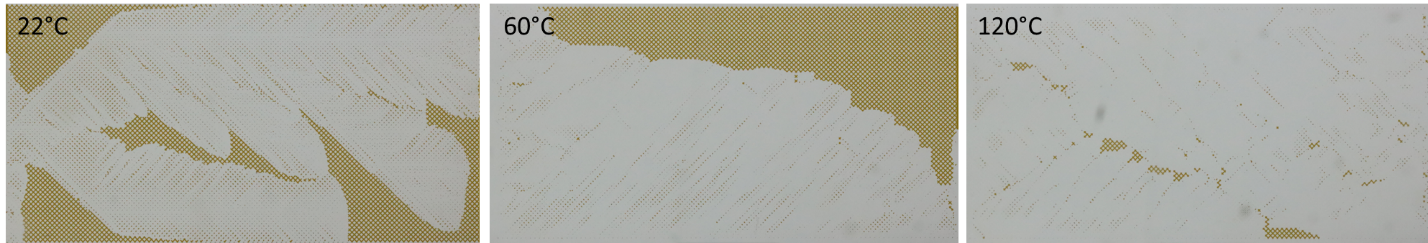


Figure S1. Pictures of crude oil A displaced by HS-Na at breakthrough at different temperatures.


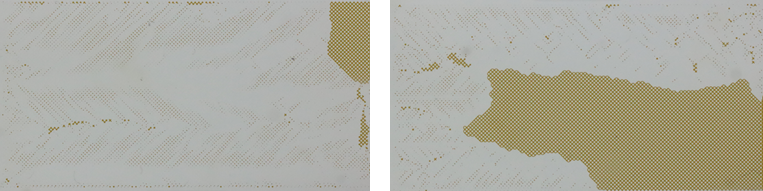


Figure S2. Different patterns of crude oil A displaced by HS-Na at breakthrough at 100°C.


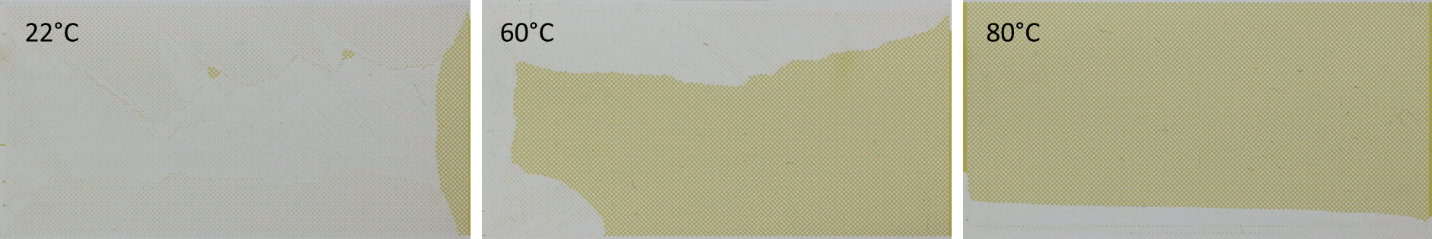


Figure S3. Pictures of crude oil C displaced by HS-Na at breakthrough at different temperatures.


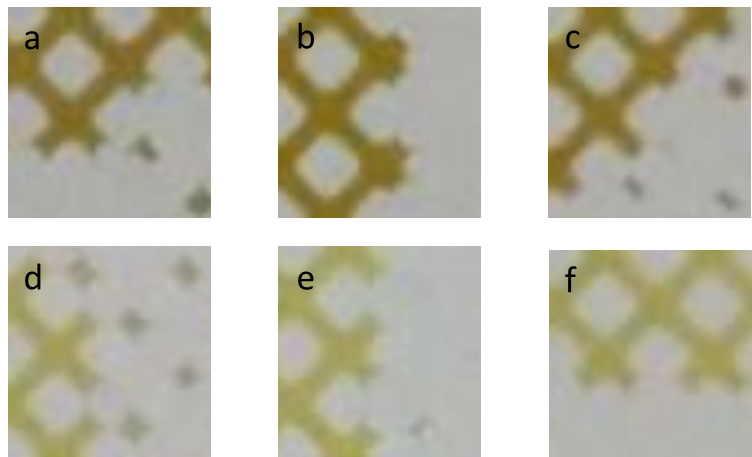


Figure S4. Non-edited close-up images of the border regions between the remaining oil and brine solution having displaced oil in the porous network for crude oil A at 22°C (a), 60°C (b) and 100°C (c) and crude oil C at 22°C (d), 60°C (e) and 80°C (f).


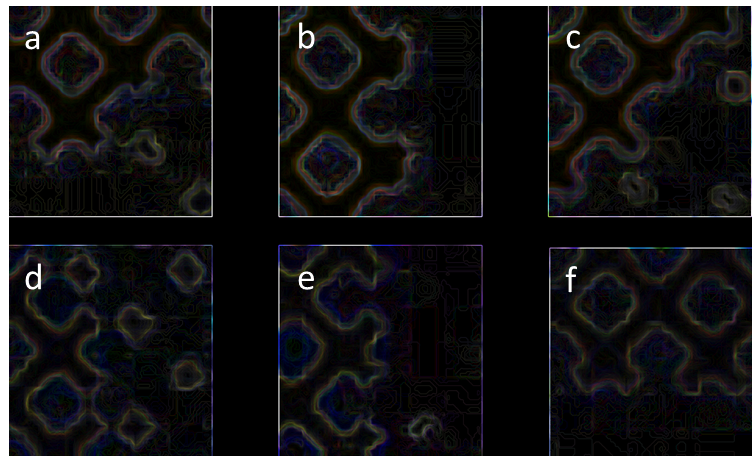


Figure S5. Processed images of the border regions, highlighting edges between the remaining oil and brine solution having displaced oil in the porous network for crude oil A at 22°C (a), 60°C (b) and 100°C (c) and crude oil C at 22°C (d), 60°C (e) and 80°C (f).


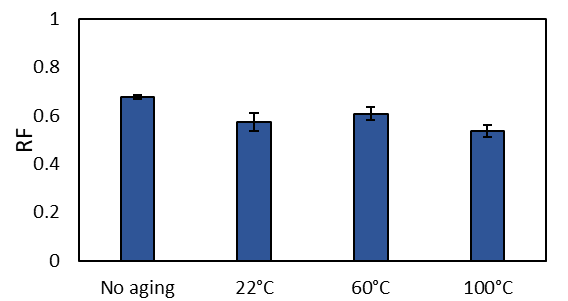


Figure S6. RF for crude oil A displaced by HS-Na at ambient temperature and pressure after being aged for 2 hours at different temperatures. A non-aged test is also presented for reference.
